# Understanding the Role of *Trichoderma reesei* Vib1 in Gene Expression during Cellulose Degradation

**DOI:** 10.3390/jof7080613

**Published:** 2021-07-29

**Authors:** Xiuzhen Chen, Bingran Song, Minglu Liu, Lina Qin, Zhiyang Dong

**Affiliations:** 1 State Key Laboratory of Microbial Resources, Institute of Microbiology, Chinese Academy of Sciences, Beijing 100101, China; chenxiuzhen@im.ac.cn (X.C.); songbingran16@mails.ucas.edu.cn (B.S.); liuminglu17@mails.ucas.ac.cn (M.L.); 2 National and Local Joint Engineering Research Center of Industrial Microbiology and Fermentation Technology, College of Life Sciences, Fujian Normal University, Fuzhou 350117, China; qinln@fjnu.edu.cn

**Keywords:** *Hypocrea jecorina*, lignocellulose, biomass, biorefinery, cellulase, transcription factor, secondary metabolism, transcriptome

## Abstract

Vib1, a member of the Ndt80/PhoG-like transcription factor family, has been shown to be essential for cellulase production of *Trichoderma reesei*. Here, we combined transcriptomic and genetic analyses to gain mechanistic insights into the roles of Vib1 during cellulose degradation. Our transcriptome analysis showed that the *vib1* deletion caused 586 genes with decreased expression and 431 genes with increased expression on cellulose. The downregulated genes were enriched for Gene Ontology terms associated with carbohydrate metabolism, transmembrane transport, oxidoreductase activity, and transcription factor activity. Of the 258 genes induced by cellulose, 229 showed no or decreased expression in Δ*vib1* on cellulose, including almost all (hemi)cellulase genes, crucial sugar transporter genes (IDs:69957, 3405), and the genes encoding main transcriptional activators Xyr1 and Ace3. Additionally, Vib1 also regulated the expression of genes involved in secondary metabolism. Further comparison of the transcriptomes of Δ*vib1* and Δ*xyr1* in cellulose revealed that the genes regulated by Vib1 had much overlap with Xyr1 targets especially for the gene set induced by cellulose, presumably whose expression requires the cooperativity between Vib1 and Xyr1. Genetic evidence indicated that Vib1 regulates cellulase gene expression partially via Xyr1. Our results will provide new clues for strain improvement.

## 1. Introduction

Lignocellulosic biomass is the most abundant source readily available that presents an enormous potential for the production of biofuels and other bio-based products [1]. Bioconversion of cellulose substrate into soluble sugars through cellulolytic enzymes represents a green, sustainable strategy for biorefinery [2,3]. The filamentous fungus *Trichoderma reesei* is one of the most extraordinary producers of cellulases and hemicellulases, and it has become a paradigm in breakdown of cellulosic biomass [4]. However, since the recalcitrance of lignocellulosic biomass to enzymatic hydrolysis leads to a requirement for large amounts of lignocellulolytic enzymes, the high cost of enzyme, which ranges from $0.23 to $0.78 per gallon of ethanol [5], is still a major bottleneck in lignocellulosic biofuel production [6,7]. Genetic engineering of *T. reesei* for enhanced enzyme production is an efficient approach to lower the contribution of enzymes to biofuel production costs. However, the progress is very slow due to our incomplete knowledge of the molecular mechanisms underlying cellulase expression. A better understanding of the regulatory network controlling cellulase production in *T. reesei* is imperative and would assist to develop new biotechnologies for cheaper production of cellulases.

The cellulase complex from *T. reesei* at least includes three types of synergistically acting enzymes: endoglucanases (EC 3.2.1.4), exoglucanases/cellobiohydrolases (EC 3.2.1.91), and beta-glucosidases (EC 3.2.1.21), whose production is regulated mainly at the transcriptional level and obligatorily requires the presence of an inducer such as cellulose, disaccharides (cellobiose, sophorose) generated from cellulose degradation, or the soluble carbon source lactose. Additionally, most *T. reesei* cellulase genes are coordinately expressed [8,9], suggesting that a tight regulatory scheme operates in this fungus, which involves the fine-tuned cooperation of the respective transcription factors [10]. Several transcription factors are implicated in this process [11], including the positive master regulator Xyr1 [12] and the major carbon catabolite repressor Cre1 [13]. The last decade of investigation has revealed novel transcription factors for cellulase expression, including positive regulators Ace3 [14], Vel1 [15], BglR [16], Vib1 [17,18], and Azf1 [19], as well as the transcription repressor Rce1 [20]. Despite substantial advancements in identifying transcription factors, our knowledge is still incomplete about their roles during cellulase induction in *T. reesei.*

Vib1, a member of the Ndt80/PhoG-like transcription factor family that participates in the regulation of various metabolism processes, including meiosis [21], biofilm formation [22], and the response to nutrient stress [23], has recently been identified as a crucial regulator of cellulase production in *T. reesei* by two research groups with different means [17,18]. Vib-1, an orthologue of Vib1 in *Neurospora crassa*, is not only required for the expression of genes necessary for programmed cell death [24], but also essential for cellulose utilization [25]. It was proposed that the effect of *N. crassa* Vib-1 on cellulase production is achieved by inhibiting Cre1, and Col26, the BglR homologue of *N. crassa*, and inducing the positive master Clr-2 [25]. However, it is not clear which genes are subjected to regulation of the *T. reesei* Vib1 during cellulose degradation and the extent of overlap between the Vib1 regulon and the Xyr1 targets. In addition, it remains unknown whether Vib1 exerts its functions through Xyr1.

Here, to determine whether the requirement for vib1 varies with the respective inducers, we investigated the cellulase production of the Δ*vib1* mutant under different carbon sources. To define the extent of the Vib1 regulon, we performed RNA-seq to assess the genome-wide gene expression differences between parent strain and Δ*vib1* grown on cellulose. In addition, to determine the genes regulated by Vib1 and Xyr1, we compared the transcriptomes of Δ*vib1* and Δ*xyr1* and mainly focused on analyzing the expression level of the Avicel regulon, the gene set that had higher expression on cellulose than under either glucose or no carbon source conditions. Moreover, we introduced an additional copy of constitutive *xyr1* expression cassette into Δ*vib1* to test whether Vib1 exerts its functions through Xyr1.

## 2. Materials and Methods

### 2.1. Strains and Cultivation Conditions

*T. reesei* strains TU6 (ATCC MYA-256) Δ*tku70*(TU6), TU6_Δ*tku70*Δ*vib1*(Δ*vib1*), vib1ce (constitutive expression of *vib1* in Δ*vib1*), TU6_Δ*tku70*Δ*xyr1*(Δ*xyr1*), and Δ*vib1::xyr1* (constitutive expression of *xyr1* in Δ*vib1*) were cultivated on liquid minimal medium (MM) without peptone described previously [8], on a rotary shaker (250 rpm), at 28 °C. The pH of MM was adjusted to 5.1 ± 0.2 with NaOH. Carbon sources were 1% (*w/v*), except that glucose was supplemented with 2% (*w/v*).

For replacement experiments [26], *T. reesei* strains were precultured in MM with glucose as sole carbon source for 30 h. Pre-grown mycelia were collected by gauze filtration and washed twice with carbon-free MM. Equal amounts of mycelia were transferred to flasks containing the appropriate carbon source (Avicel cellulose, lactose, cellobiose, and glucose) or no carbon source added and continued cultivation. At indicated time points, the cultures were sampled and centrifuged at 12,000 rpm, for 10 min, at 4 °C. The culture supernatants were then used for cellulase activity and protein concentration assays, whereas the harvested mycelia were used for biomass determination or total RNA isolation.

### 2.2. Enzyme Activity and Protein Concentration Assays

The filter paper hydrolyzing activity (FPase) and endo-beta-1,4-glucanase (CMCase) activity in culture supernatants were measured according to the International Union of Pure and Applied Chemistry (IUPAC) standard [27]. The extracellular protein concentration was determined by the Bradford method (Sangon Biotech Co., Ltd., Shanghai, China).

### 2.3. RNA Isolation, Library Preparation, RNA Sequencing

Mycelia were sampled at 8 h after shifting to MM containing Avicel cellulose, glucose, or no carbon source. Total RNA was extracted with TRIzol reagent (Invitrogen Life Technologies, Carlsbad, CA, USA), following the manufacturer’s instructions. RNA integrity and purity were monitored on 1% agarose gels. Additional quality assessments were performed with the RNA Nano 6000 Assay Kit of the Bioanalyzer 2100 system (Agilent Technologies, Santa Clara, CA, USA) and the NanoPhotometer^®^ spectrophotometer (IMPLEN, Los Angeles, CA, USA). RNA concentration was measured by using a Qubit^®^ RNA Assay Kit in a Qubit^®^ 2.0 Fluorometer (Life Technologies, Carlsbad, CA, USA).

The libraries were generated by using the NEBNext^®^ Ultra™ RNA Library Prep Kit for Illumina^®^, following the manufacturer’s recommendations, and subjected to sequencing on an Illumina HiSeq platform with paired-end reads. The RNA-seq raw data are available at the SRA web site (https://www.ncbi.nlm.nih.gov/sra, accessed on 28 March 2021), under accession number SRP312496.

### 2.4. RNA-Seq Data Analysis

Clean data were obtained by removing reads containing adapter, poly-N and low-quality reads from raw data through in-house Perl scripts. Then the clean reads were mapped to the transcripts from the reference genome (https://genome.jgi.doe.gov/Trire2/Trire2.home.html, accessed on 26 January 2018), using Hisat2 v2.0.4. HTSeq v0.9.1 was used to count the reads numbers mapped to each gene and calculate FPKM (expected number of fragments per kilobase of transcript sequence per millions base pairs sequenced) [28]. Differential expression analysis of two conditions or groups was performed with the DESeq R package (1.18.0), with read counts as inputs. Genes with an adjusted *p*-value < 0.05 and |log_2_fold change| ≥ 1 were assigned as differentially expressed. Gene Ontology (GO) enrichment analysis of differentially expressed genes was implemented by the GOseq R package, in which gene length bias was corrected. GO terms with corrected *p*-value less than 0.05 were considered significantly enriched by differentially expressed genes.

### 2.5. Real-Time Quantitative PCR (RT-qPCR)

DNase I-treated total RNA was used to synthesize first-strand cDNA according to the Invitrogen Superscript III first-strand synthesis kit. Except for *rpl6e*, which was used as a reference gene [29], RT-qPCR assays were performed as described by Chen et al. [30].

### 2.6. Construction of the T. reesei Mutants

For the recyclable use of the marker gene *pyr4* (ID 74020), a 3.7 kb *pyr4* blaster cassette with a loopout fragment was constructed as described [31]. In brief, the primer pairs Fpyr4-DR/Rpyr4-DR and Fpyr4/Rpyr4 were designed to amplify direct repeats (1.0 kb upstream of the *pyr4* start codon) and 2.3 kb of the *pyr4* expression cassette that consisted of direct repeats, *pyr4* coding regions, and *pyr4* terminators, respectively. The two purified PCR products were digested with *HindIII*/*NdeI* and *NdeI*/*BamHI* and ligated with *HindIII*/*BamH1*-digested pBluescript SK-plus (Stratagene), resulting in the pPYR4 plasmid. The 3.7 kb *pyr4* blaster cassette was released through the digestion of plasmid pPYR4 with *HindIII*/*BamHI* and used for subsequent plasmid construction.

The Δ*vib1* mutant was generated by electroporating the uridine auxotrophic strain TU6_Δ*tku70* with the *vib1* gene deletion cassette, which contains 1.8 kb of 5′ and 3′ flanking regions of the open reading frame of *vib1* and a 3.7 kb of *pyr4* blaster cassette. The Δ*xyr1* mutant was obtained in the same manner, except that the 5′ and 3′ flanking regions of the *xyr1* gene were employed.

The vib1ce strain, in which *vib1* was constitutively expressed in Δ*vib1*, was constructed by transforming the uridine auxotrophy strain Δ*vib1* with a DNA fragment containing *A. nidulans* glyceraldehyde-3-phosphate dehydrogenase (GenBank: M33539.1) promoter, the open reading frame and terminator region of the *vib1* gene, and 2.7 kb *pyr4* expression cassette and 1.8 kb of sequence downstream of the *pyr4* stop codon used as homologous arms for targeted integration in the *pyr4* locus. The *xyr1* expression cassette targeting the *pyr4* locus was constructed in the same way, except that the coding and terminator regions of *xyr1* gene were used. The *xyr1* expression cassette was transformed into Δ*vib1* to obtain Δ*vib1*::*xyr1* strain.

The protocol for electroporation of *T. reesei* was performed according to the method described by Schuster et al. [29], except that a BIO-RAD Gene Pulser Xcell electroporation system was used in our study. The PCR method was used to confirm targeted integration of gene deletion or expression cassettes [32]. The copy number of the cassette integrated in the genome was determined by quantitative real-time PCR on the genomic DNA, as previously described [33].

All primers used in this study are listed in Supplementary Materials Table S1.

## 3. Results

### 3.1. Vib1 Is Required for Cellulolytic Enzyme Production Independent of Carbon Sources

**Figure 1:**
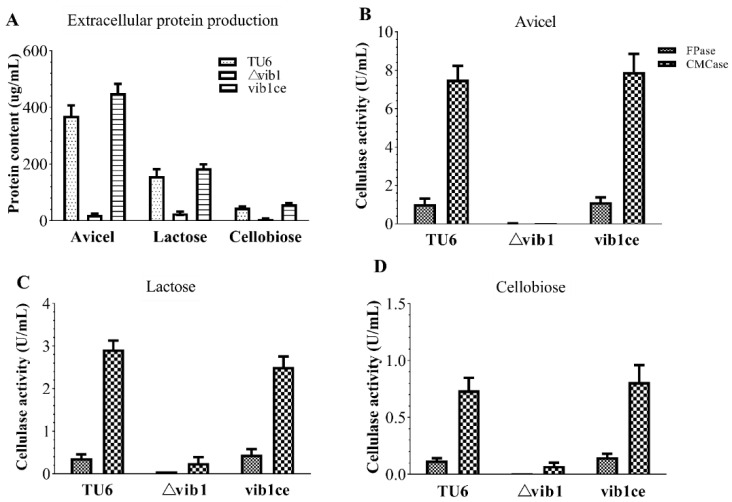
The effect of Vib1 on extracellular protein production and cellulase activity under different carbon sources. Parent strain TU6 and the *vib1* deletion mutant (Δvib1), together with the vib1 constitutive expression strain (vib1ce), were precultured in glucose-containing MM for 30 h; thereafter, the pre-grown mycelia were equally shifted to MM with Avicel cellulose, lactose, or cellobiose as the sole carbon source and continued cultivation for 120 h. The supernatants were collected for measuring extracellular protein content (**A**) and the volumetric cellulase activity (**B**–**D**). The values were means from three biological replicates. Error bars denoted standard deviations.

The *vib1* deletion mutant (Δ*vib1*) failed to provoke cellulase formation under cultivation conditions supplemented with a mixture of Solka-Floc cellulose and lactose as carbon sources [17]. We wondered whether the *vib1* deletion causes differential responses to the respective inducers; thus, we investigated the cellulase production of the Δ*vib1* mutant under liquid MM with crystalline cellulose (Avicel PH-101, Sigma-Aldrich, St. Louis, MI, USA), lactose, or cellobiose as the sole carbon source. Irrespective of inducer strengths, Δ*vib1* failed to produce extracellular protein on Avicel, lactose, or cellobiose (Figure 1), which is consistent with the results that almost no cellulase activity indicated by filter paper activity and CMCase activity was detected in strains lacking *vib1* (Figure 1). The Δ*vib1* mutant showed defect growth on Avicel but not on lactose and cellobiose (Supplementary Materials Figure S1). The ectopic integration of a copy of *vib1* expression cassette driven by glyceraldehyde-3-phosphate dehydrogenase promoter from *Aspergillus nidulans* completely restored the growth of the Δ*vib1* mutant on Avicel, extracellular protein production, and cellulase activity.

### 3.2. Comparative Transcriptome Analysis of Parent Strain and the Δvib1 Mutant

To comprehensively understand the exact role of Vib1 in cellulose degradation, we utilized next-generation RNA sequencing to profile genome-wide mRNA abundances when the Δ*vib1* mutant and parent strain were shifted to Avicel, glucose, or carbon-free medium for 8 h, following 30 h of pre-cultivation in glucose. RNA samples of three biological replicates from each condition were used for library preparation and sequencing, resulting in 15 sets of RNA-seq data. The data from three replicates of each condition showed a high Pearson correlation (Supplementary Materials Data S1).

For the parent strain, a comparative transcriptional profiling analysis between Avicel and no carbon source showed that 707 genes were differentially expressed, including 371 genes upregulated and 336 genes downregulated upon exposure to Avicel (Supplementary Materials Data S2). Only in the 371 upregulated gene set, there were enriched GO terms associated with hydrolase activity (corrected *p*-value: 1.33 × 10**^−^**^17^), carbohydrate metabolism (corrected *p*-value: 1.15 × 10**^−^**^15^), cellulose binding (corrected *p*-value: 1.14 × 10**^−^**^7^), and polysaccharide binding (corrected *p*-value: 7.48 × 10**^−^**^7^) (Supplementary Materials Figure S2). Of the 371 genes highly expressed on Avicel, 258 genes accumulated much more transcripts on Avicel than on glucose or no carbon (Supplementary Materials Figure S3), which were assigned as the “Avicel regulon” (Supplementary Materials Data S2). The Avicel regulon contained 18 genes involved in cellulose degradation, including major cellulase genes and nonenzymatic cellulose attacking protein encoding genes; 23 characterized or predicted hemicellulase genes; 14 genes encoding predicted proteins with signal peptides. Additionally, included in the Avicel regulon were 22 genes encoding sugar transporters, including recently identified lactose permease Ctr1 (ID 3405) [32,34], mannose/cellobiose/xylose transporter (ID 69957) [35]. Additionally, the genes especially induced by cellulose contained 16 transcription factors, including Xyr1, Ace3, AmyR, and *N. crassa* cellulase regulator Clr-2 orthologue (IDs: 122208, 77513, 55105 and 26163). Genes involved in protein folding and modification were also included in the Avicel regulon. According to our conservative differential expression analysis (adjusted *p*-value < 0.05 and |Log_2_fold change| ≥ 1), *vib1*(ID 54675) was not grouped into the Avicel regulon, but the mRNA level of *vib1* was relatively higher on Avicel than either on no-carbon or glucose conditions.

**Figure 2:**
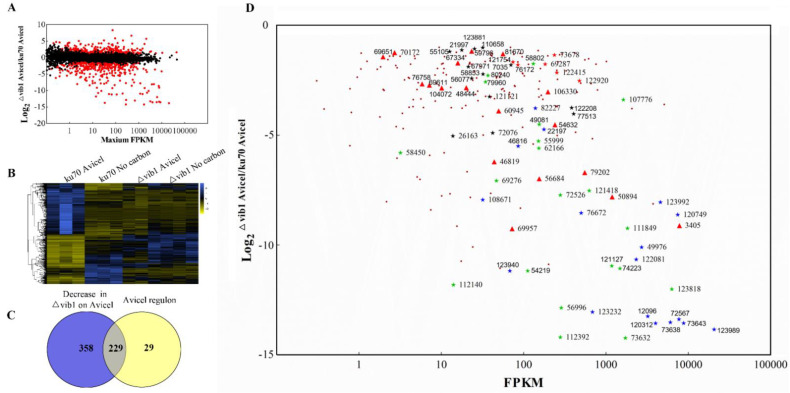
Transcriptional response of the Δ*vib1* mutant to cellulose induction. (**A**) Genome-wide analysis of the influence of VibIB1 on the transcriptomes of *T. reesei* on Avicel. Log_2_ ratio of Δ*vib1*/TU6 on Avicel vs. maximum FPKM in either condition. Genes exhibiting differential expression (|Log_2_fold change| ≥ 1 and adjusted *p*-value < 0.05) are indicated with a red dot. (**B**) Hierarchical clustering of expression levels in Δ*vib1* and TU6 for 707 genes differentially expressed in TU6 on Avicel cellulose and no carbon. Data from three biological replicates were included, and the expression level of gene was indicated with log_10_ FPKM. (**C**) Venn diagram of genes downregulated in the Δ*vib1* mutant on Avicel cellulose compared to the Avicel regulon. (**D**) Fold change and expression level of the gene set within the Avicel regulon whose expression was downregulated in Δ*vib1* on Avicel cellulose. Log_2_ ratio of Δ*vib*1/TU6 read-count vs. FPKM in TU6 on Avicel was plotted. Genes encoding cellulase (★), hemicellulase (★), transcriptional factor (★), sugar transporter (▲), and secretory pathway component (★) are labeled with protein ID and indicated by the different symbols and colors. The other genes are indicated by red dot (.).

To gain insights into the extent to which Vib1 affects gene expression on Avicel, we compared the transcriptomes of the *vib1* deletion and parent strains under Avicel cultivation condition, and the expression of the abovementioned 707 differentially expressed genes in Δ*vib1* on Avicel vs. on no carbon. The absence of *vib1* caused downregulation of 586 genes and upregulation of 431 genes in relative to parent strain on Avicel (Figure 2 and Supplementary Materials Data S3). The downregulated 586 genes were named as the “Vib1-dependent gene set”, which was enriched in functional categories, such as carbohydrate metabolism (corrected *p*-value: 1.36 × 10^−23^), transmembrane transport (corrected *p*-value: 5.58 × 10^−19^), hydrolase activity acting on glycosyl bonds (corrected *p*-value: 6.30 × 10^−29^), oxidoreductase activity (corrected *p*-value: 4.27 × 10^−16^), and RNA polymerase II transcription factor activity (corrected *p*-value: 7.15 × 10^−9^) (Supplementary Materials Figure S4A). For the upregulated genes, the most enriched functional categories were single-organism metabolic process (corrected *p*-value: 1.47 × 10^−5^), oxidation-reduction process (corrected *p*-value: 4.96 × 10^−10^), and transmembrane transport (corrected *p*-value: 2.86 × 10^−5^) (Supplementary Materials Figure S4B). For the 707 genes differentially expressed on Avicel and no carbon in the parent strain, the Δ*vib1* mutant on Avicel showed almost the same expression profile as that on the no-carbon source (Figure 2 and Supplementary Materials Data S2), indicating that the Δ*vib1* mutant failed to respond to the presence of cellulose in the environment, which supports the notion that sensing and responding to nutritional status is one of the ancestral roles for the Ndt80 family members [23].

### 3.3. Analyses of the Vib1-Regulated Genes

Of the 586 Vib1-dependent genes, about 89% (229 genes) of the Avicel regulon showed no or reduced gene expression on Avicel in Δ*vib1* when compared to parent strain (Figure 2 and Supplementary Materials Data S3), which comprise almost all (hemi)cellulose-degrading genes except *cel3e* within the Avicel regulon and 18 sugar transporter genes, including ID 69957 and ID 3405. Additionally, 13 out of 16 transcription-factor-encoding genes in the Avicel regulon were significantly downregulated in the Δ*vib1* mutant, including *xyr1*, *ace3*, *amyr*, and the *N. crassa clr-2* orthologue (Figure 2). Vib1 also had positive effects on the transcriptional levels of genes involved in protein folding and modification (IDs: 122415, 122920, 60085, and 73678). The remaining 29 genes within the Avicel regulon mainly encode hypothetical proteins. These data indicated that Vib1 is a crucial regulator of the Avicel regulon in *T. reesei.* Besides the Avicel regulon, Vib1 also influenced the expression levels of genes encoding the transcription factors AreA (ID: 76817) and the *N. crassa* Clr-1 orthologue (ID: 27600). It shall be noted that reduced expression of *cre1* (log_2_fold change = −0.91818, *p* = 0.023) was observed in Δ*vib1*.

Our transcriptome analysis also pointed to a regulatory role of Vib1 in secondary metabolism. Sorbicillin, a typical yellow pigment secreted by fungi, including *Trichoderma* [36,37,38], is a hexaketide secondary metabolite with diverse bioactivities [39,40] The sorbicillin (SOR) gene cluster comprises two polyketide synthases, Pks11/Sor1 (ID 73618) and Pks10/Sor2 (ID: 73621); two auxiliary modifiers (IDs: 73623 and 73631); one transporter (ID: 43701); and two yellow pigment regulators, Ypr1 and Ypr2 (IDs: 102499 and 102497) [36,41]. Our results showed that all genes within the SOR cluster displayed enhanced transcript accumulation on Avicel in Δ*vib1* (Table 1), with *sor3* having the highest fold change (log_2_fold change = 8.3199). Besides the SOR gene cluster, Vib1 also affected the expression of three polyketide synthase genes (IDs: 65172, 82208, and 59482), four nonribosomal peptide synthase (NRPS) genes (IDs: 81014, 123786, 68204, and 69946), and one NRPS/PKS fusion gene (ID: 58285). Additionally, Vib1 negatively regulated the expression level of the transcription factor Vel1, a regulator of cellulase gene expression, development, and secondary metabolism biosynthesis [15].

### 3.4. The Vib1-Dependent Gene Set Considerably Overlaps with Xyr1 Targets

**Figure 3:**
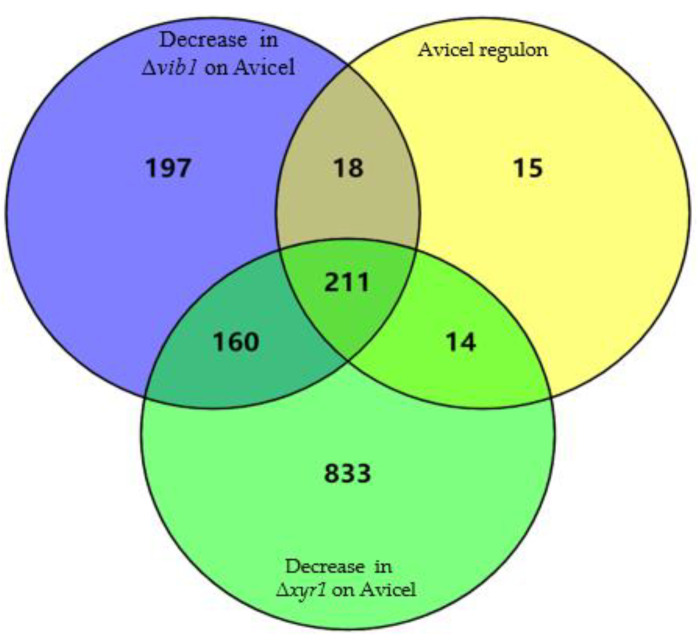
Venn diagram of genes downregulated in Δ*vib1* and Δ*xyr1* on Avicel compared to the Avicel regulon.

For cellulase and extracellular protein production on cellulose, Vib1 demonstrated almost the same output as the cellulase transcriptional regulator Xyr1, suggesting that Vib1 may share regulatory targets with Xyr1. To test this hypothesis, we also performed transcriptome sequencing of the Δ*xyr1* mutant on cellulose in parallel with the Δ*vib1* mutant and parent strain. The deletion of *xyr1* caused 2064 genes differentially expressed when compared with parent strain on Avicel, including 1218 downregulated genes and 846 upregulated genes (Supplementary Materials Data S4). The Vib1-dependent 586-gene set overlapped with Xyr1 targets by 371 genes. Of the 229 genes within the Avicel regulon which were downregulated in Δ*vib1*, 211 genes showed decreased or no expression in Δ*xyr1* on Avicel (Figure 3).

The overlapped 211 genes included almost all (hemi)cellulose-degrading genes, except for *cel3e*, within the Avicel regulon; 14 sugar transporter genes, including ID 69957 and ID 3405; and 11 (putative) transcription factor-encoding genes, including ace3 and theclr−2 homologue (Table 2). Genes involved in posttranslational modification, intracellular trafficking, and secretion (e.g., ER chaperone Bip1, protein disulfide isomerase Pdi1, DnaJ superfamily molecular chaperone, COPII vesicle protein, ER-resident chaperone calnexin, alpha -1,2-mannosidase, alpha-mannosyltransferase, glucosidase II catalytic (alpha) subunit GII, and secretory pathway protein Ysy6) (Supplementary Materials Table S3) were also regulated in common by Vib1 and Xyr1. Of note, over 50% of 211 genes were affected by Vib1 and Xyr1 to a similar degree, suggesting that the cooperativity between Vib1 and Xyr1 might be required for the expression of these genes.

### 3.5. Constitutive Expression of xyr1 Partially Restored the Cellulase Production Ability of the Δvib1 Mutant

**Figure 4:**
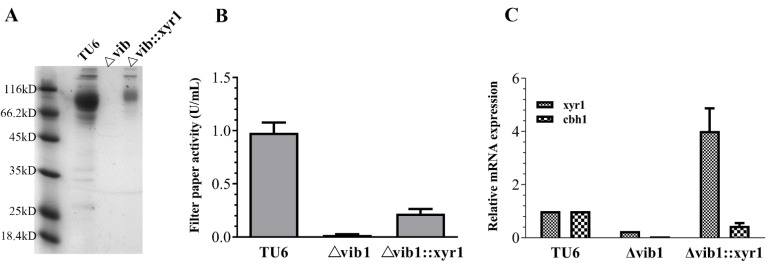
Constitutive expression of ***xyr1*** partially restored cellulase production of the Δ***vib1*** mutant on Avicel. The secretomes (**A**) and cellulase activity (**B**) of the culture supernatant in TU6, Δ*vib1*, and Δ*vib1*::*xyr1* 120 h after transfer to Avicel cellulose from glucose. (**C**) RT-PCR measurement of *xyr1* and *cbh1* expression in TU6 versus Δ*vib1* and Δ*vib1*::*xyr1* 24 h after transfer to Avicel cellulose from glucose. Expression level was normalized to the parent strain TU6.

The result that the *vib1* deletion significantly reduced the transcriptional level of *xyr1* on cellulose prompted us to ask whether Vib1 acts through Xyr1 to regulate cellulase gene expression. If Xyr1 is subjected to the direct control of Vib1, the constitutive expression of *xyr1* gene theoretically should bypass the requirement of Vib1. To test this hypothesis, we constructed strain Δ*vib1*::*xyr1*, in which an additional copy of the *xyr1* constitutive expression cassette was integrated into the genome of Δ*vib1*. As shown in Figure 4, constitutive expression of *xyr1* only partially restored the cellulolytic capacity of Δ*vib1*; however, the mRNA level of *xyr1* in the Δ*vib1*::*xyr1* mutant was higher than that in parent strain. These results suggested that Vib1 acts at least in part via Xyr1 to regulate cellulase biosynthesis; on the other hand, the exertion of the Xyr1 function likely depends on the involvement of Vib1 or other additional factors.

## 4. Discussion

In the present study, we demonstrated that the Δ*vib1* mutant abolished cellulase production irrespective of inducer strength and displayed defect growth on Avicel but not on lactose and cellobiose. By RNA-seq, we analyzed the genes regulated by Vib1 under Avicel cultivation conditions and identified the core gene set commonly regulated by Vib1 and Xyr1. By genetic analysis, we showed that Vib1 controls cellulase expression partially via Xyr1.

Our transcriptome comparison between parent strain and Δ*vib1* showed that the *vib1* deletion significantly reduced the expression level of *xyr1*, *ace3, cre1,* and the *N. crassa clr-1* and *clr-2* homologues, but exerted no effect on the mRNA level of *bglR* on Avicel. Similarly, the expression of *bglR* on lactose was also not affected by Vib1 [17]. This is different from the case in *N. crassa*, where functional loss of *N. crassa* Vib1 resulted in significantly reduced mRNA levels of the transcription factor Clr2 essential for cellulase induction [42] but increased transcriptional levels of the genes encoding carbon catabolite repressor Cre1 and the *T. reesei* BglR orthologue Col-26 critical for glucose sensing/metabolism [25]. The reason for this phenomenon is the difference of transcription machinery between *T. reesei* and *N. crassa* [43]. Therefore, despite functional conservation in cellulase production, the regulatory circuit of Vib1 was rewired in *T. reesei*.

Previous studies revealed that the transcription factor Ace3 is essential for the expression of cellulase genes [14], which governs cellulase activity through Xyr1 and the cellulose response transporter Crt1 [34]. Here, we showed that the deletion of *vib1* or *xyr1* significantly reduced the transcript accumulation of *ace3*. Meanwhile, Vib1 and Xyr1 positively regulated each other irrespective of the extent. Additionally, by analyzing the gene expression pattern of the Avicel regulon in Δ*vib1* and Δ*xyr1* on cellulose, we found that there was considerable overlap between the genes regulated by Vib1 and Xyr1. Taken together, it is possible that Vib1, Xyr1, and Ace3 constitute a core regulatory circuit and may depend on each other to achieve individual functions. Alternatively, the effect of Vib1 on cellulase expression may be achieved through activating Ace3, thus facilitating the interaction of Ace3 with Xyr1 or other factors.

There are several lines of evidence pointing to the crosstalk between plant cell wall deconstruction enzyme expression and secondary metabolism biosynthesis in *T. reesei* [37,44,45,46,47,48], which involves methyltransferase Lae1, YPK1-type kinase Usk1, catalytic subunit of protein kinase A (PKAc1), and transcription factors (Vel1, Cre1, Xpp1, and Ypr2). It has been demonstrated that Cre1, Usk1, PKAc1, and Xpp1 control the expression of the SOR cluster, including the transcription factors Ypr1 and Ypr2 [37,45,47,48]. Our results indicated that, besides cellulase biosynthesis, Vib1 controls the expression of the SOR gene cluster; however, Vib1 had no effect on the mRNA levels of Usk1, PKAc1, and Xpp1, indicating that Vib1 might act downstream of the three regulators. This requires further investigation. In addition to the SOR cluster, we found that VIB1 also regulated the expression of three PKS genes, four NRPS genes, one NRPS/PKS fusion gene, and the global secondary metabolism regulator Vel1. These findings suggest that Vib1 might exert a broad influence on secondary metabolism in *T. reesei*.

## 5. Conclusions

This study showed that Vib1 was involved in cellulose degradation through the control of multiple gene expression beyond carbohydrate metabolism. The set of 586 Vib1-dependent genes overlapped with Xyr1 targets by 371 genes, including 211 genes within the Avicel regulon. The overlapped 211 genes included almost all (hemi)cellulose-degrading genes, except for cel3e; and characterized crucial sugar transporter- and transcription factor-encoding genes, which may constitute the backbone of the whole cellulose degradation system. We propose that their expression may be dependent on the cooperativity between Vib1 and Xyr1. The result that constitutive expression of *xyr1* only partially restored the cellulase production ability of the Δvib1 mutant, to some extent, showed that the implementation of the Xyr1 function required the involvement. Further research is needed to test this hypothesis. Taken together, the present work has revealed new aspects of cellulase expression regulation in *T. reesei*. Such knowledge has important implications for the improvement of cellulase production by *T. reesei*.

## Figures and Tables

**Table 1:** Vib1 affects the expression of the SOR cluster and other secondary metabolism-associated genes on Avicel.

Description	Protein ID	Log_2_ (Δ*vib1*/TU6)	Adjusted *p*-Value
Polyketide synthase Pks11S/Sor1	73618	4.324	1.89 × 10^−19^
Polyketide synthase Pks10S/Sor2	73621	3.941	1.46 × 10^−11^
FAD-dependent monooxygenaseSor3	73623	8.320	3.04 × 10^−10^
FAD/FMN-containing dehydrogenase/Sor4	73631	1.844	0.0021573
MFS multidrug-resistance transporter	43701	4.243	1.36 × 10^−33^
Transcription factor Ypr1	102499	2.513	0.00012598
Transcription factor Ypr2	102497	2.788	6.22 × 10^−16^
Polyketide synthase Pks1	65172	2.773	0.01383
Polyketide synthase Pks4	82208	−2.448	0.013456
Polyketide synthase Pks5	59482	−1.770	6.83 × 10^−7^
Non-ribosomal peptide synthases (NRPS)	81014	−1.154	0.03429
Non-ribosomal peptide synthases (NRPS)	123786	−1.192	0.0049844
Non-ribosomal peptide synthases (NRPS)	68204	−1.801	2.8× 10^−7^
Non-ribosomal peptide synthases (NRPS)	69946	−1.449	6.04× 10^−5^
NRPS/PKS hybrid	58285	1.449	0.0011556
Transcription factor Vel1	122284	1.473	3.32× 10^−5^

**Table 2:** Fold change of genes encoding cellulase, hemicellulase, sugar transporter, and transcription factor of the Avicel regulon in Δ*vib1* and Δ*xyr1* on Avicel.

Category	Description	Protein ID	Log2 (Δvib1/TU6)	Log2 (Δxyr1/TU6)
Cellulase				
	Endo-beta-1,4-glucanase Egl2/Cel5a	120312	−13.571	−13.839
	Endo-beta-1,4-glucanase Egl4/Cel61a	73643	−13.566	−13.757
	Cellulose-binding protein Cip1	73638	−13.531	−13.627
	Cellobiohydrolase I CBHI/Cel7a	123989	−13.850	−13.508
	Cellobiohydrolase II CBHII/Cel6a	72567	−13.386	−13.504
	Endo-beta-1,4-glucanase Egl4/Cel61b	120961	−13.254	−13.140
	Endo-beta-1,4-glucanase Egl3/Cel12a	123232	−13.059	−12.674
	Endo-beta-1,4-glucanase Egl1/Cel7b	122081	−10.661	−11.889
	Endo-beta-1,4-glucanase Egl5/Cel45a	49976	−10.107	−10.585
	Cellulose-binding protein Cip2	123940	−11.185	−10.497
	Non-catalytic module family expansion	123992	−8.059	−9.543
	Beta-glucosidase Bgl1/Cel3a	76672	−8.548	−8.500
	Beta-glucosidase Bgl2/Cel1a	120749	−8.625	−8.165
	Cand Beta-glucosidase Bgl3f	108671	−7.950	−7.413
	Beta-glucosidase Cel3d	46816	−5.502	−6.768
	Cand Beta-glucosidase Cel1b	22197	−4.737	−4.342
	Beta-glucosidase Cel3c	82227	−3.772	−3.146
Hemicellulase				
	Beta-galactosidase Bga1	80240	−2.282	−2.036
	Endo-beta-1,4-xylanase Xyn1	74223	−11.073	−15.128
	Cand endo-beta-1,4-xylanase Xyn5	112392	−14.207	−14.367
	Acetyl xylan esterase Axe1	73632	−14.242	−14.178
	Beta-xylosidase Bxl1	121127	−10.959	−12.475
	Beta-mannanase Man1	56996	−12.868	−12.387
	Endo-beta-1,4-xylanase Xyn2	123818	−12.017	−12.201
	Acetyl xylan esterase	54219	−11.187	−11.586
	Endo-beta-1,4-xylanase Xyn4	111849	−9.243	−10.847
	Alpha-glucuronidase Glr1	72526	−7.733	−10.480
	Acetyl esterase Aes1	121418	−7.528	−9.969
	Cand alpha-L-arabnofuranosidase Abf2	76210	−7.841	−8.324
	Endo-beta−1,4-xylanase Xyn3	120229	−7.677	−7.868
	Cand.endo-beta 1,4-xylanase	69276	−7.079	−7.685
	Cand exo-polygalacturonase	112140	−11.818	−7.545
	Cand Beta-xylosidase Xyl3b	58450	−5.803	−6.661
	Alpha-galactosidase	55999	−5.270	−6.167
	NAD (P)H-dependent D-xylosereductase Xyl1	107776	−3.387	−5.771
	Beta-mannosidase	62166	−5.597	−5.429
	Cand.beta-xylosidase/alpha-L-arabinofuranosidase	3739	−3.344	−4.965
	Xyloglucanase Cel74a	49081	−4.508	−4.639
	Cand endo-polygalacturonase	103049	−2.920	−3.504
	Cand.endo-beta 1,6-galactanase	110894	−2.663	−3.022
Sugar transporter				
	Mannose/cellobiose/xylose transporter	69957	−9.266	−10.316
	Lactose permease Crt1	3405	−9.124	−8.166
	MFS permease	50894	−7.813	−7.923
	Putative mono- or disaccharide transporters	56684	−6.986	−7.209
	MFS permease	46819	−6.216	−6.713
	Putative mono- or disaccharide transporter	79202	−6.707	−5.783
	MFS permease	54632	−4.525	−4.246
	L-arabinose isomerase	106330	−3.025	−2.557
	MFS permease	69611	−2.721	−2.523
	MFS maltose permease	48444	−2.834	−2.469
	MFS permease, hexose transporter	104072	−2.850	−2.403
	L-arabinose isomerase	60945	−3.894	−2.370
	MFS maltose permease	76758	−2.664	−1.852
	MFS L-fucose permease	67334	−1.709	−1.168
Transcription factor				
	Zn2Cys6 transcriptional regulator Xyr1	122208	−3.757	−12.387
	N-terminal binuclear Zn cluster-containing protein	72076	−4.898	−4.959
	Zn2Cys6 transcriptional regulator Clr2	26163	−5.037	−4.155
	C2HC transcriptional regulator Ace3	77513	−4.033	−3.737
	Fungal transcriptional regulatory protein	121121	−3.248	−2.913
	Fungal transcriptional regulatory protein	56077	−2.414	−2.662
	N-terminal binuclear Zn cluster-containing protein	123881	−1.065	−1.817
	ZF-MYND like protein	67971	−1.879	−1.775
	Predicted protein Myb	58853	−2.220	−1.635
	Zn2Cys6 transcriptional regulator	70351	−1.803	−1.513
	Zn2Cys6 transcriptional regulator AmyR	55105	−1.193	−1.030

## Data Availability

The RNA-seq raw data are available at the SRA website (https://www.ncbi.nlm.nih.gov/sra, accessed on 28 March 2021) under accession number SRP312496.

## Supplementary Materials

The following are available online at https://www.mdpi.com/article/10.3390/jof7080613/s1. Figure S1: Mycelial biomass accumulation of the vib1 deletion strain TU6_Δ*tku70*Δ*vib1* (Δ*vib1*) relative to the parent strain TU6_Δtku70 (TU6) and the vib1 constitutive expression strain vib1ce. Figure S2: Genome-wide analysis of transcriptional induction by Avicel cellulose in *T. reesei.* Figure S3: Hierarchical cluster of expression levels in TU6 on glucose, no-carbon, and Avicel cellulose for 707 genes differentially expressed on Avicel cellulose and no carbon 8 h after transfer. Figure S4. Gene Ontology (GO) enrichment analysis of genes differentially expressed in Δ*vib1* on Avicel as compared to TU6 on Avicel 8 h after transfer from glucose. The most enriched GO terms for downregulated genes (A) and upregulated genes (B) in the Δvib1 mutant. The GO items with adjusted *p*-value < 0.05 are indicated with the symbol “*”. Data S1: A summary of transcriptome data. Data S2: The 707 differentially expressed genes in TU6 exposed to Avicel in relative to no carbon and their expression level and fold change in Δ*vib1*. Data S3: The genes regulated by VIB1. Data S4: Gene list regulated by XYR1 on Avicel. Table S1: Oligonucleotides used in this study.
