# Supplementary material for: Understanding the Role of Trichoderma reesei Vib1 in Gene Expression during Cellulose Degradation
Source: J Fungi (Basel). 2021 Jul 29;7(8):613. doi: 10.3390/jof7080613 (PMC8397228; doi:10.3390/jof7080613)
Supplement: Supplementary file 1 [file jof-07-00613-s001.zip › Supplementary Materials/Figure 4A.pdf]

A

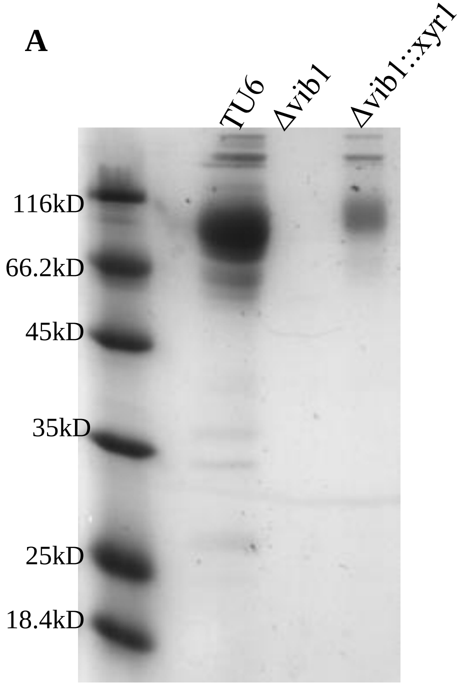

Figure 4. The secretomes of the culture supernatant in TU6, △vib1 and △vib1::xyr1 120 h after transfer to Avicel cellulose from glucose.
