# Supplementary figures and images for: Understanding the Role of Trichoderma reesei Vib1 in Gene Expression during Cellulose Degradation
Source: J Fungi (Basel). 2021 Jul 29;7(8):613. doi: 10.3390/jof7080613 (PMC8397228; doi:10.3390/jof7080613)

**A**

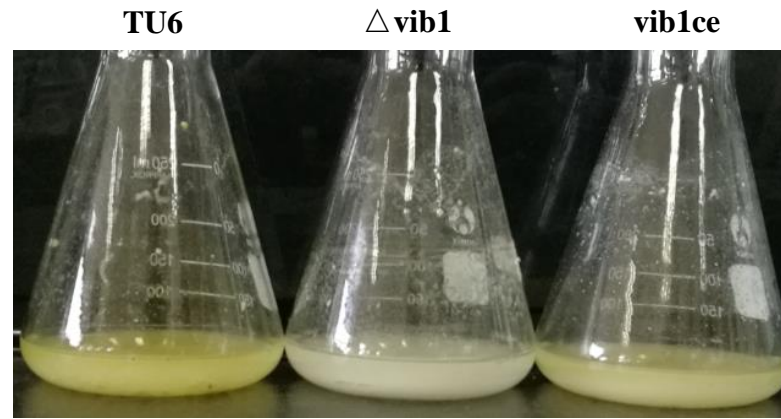

**B**

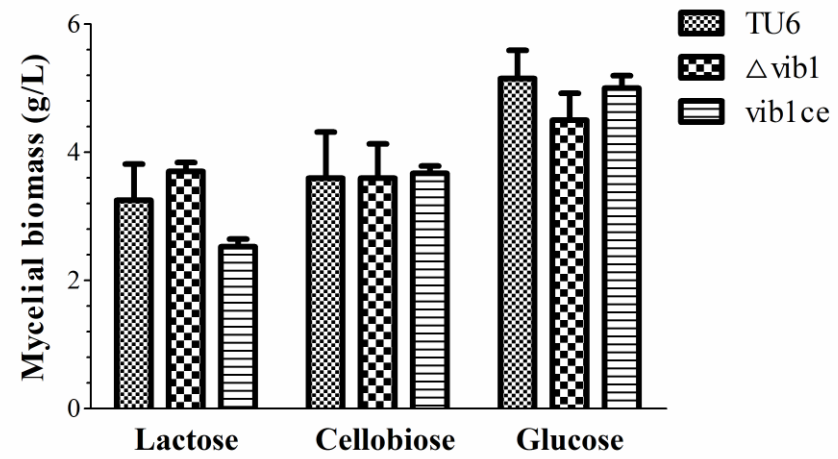

Supplement: Supplementary file 1 [file jof-07-00613-s001.zip › Supplementary Materials/Figure S1.pdf]

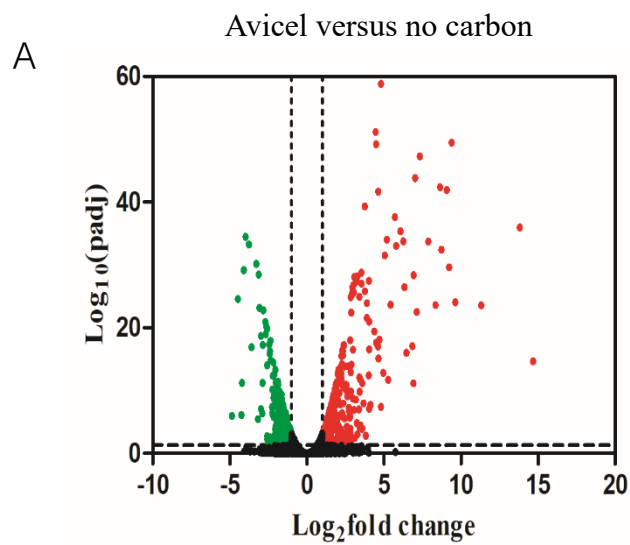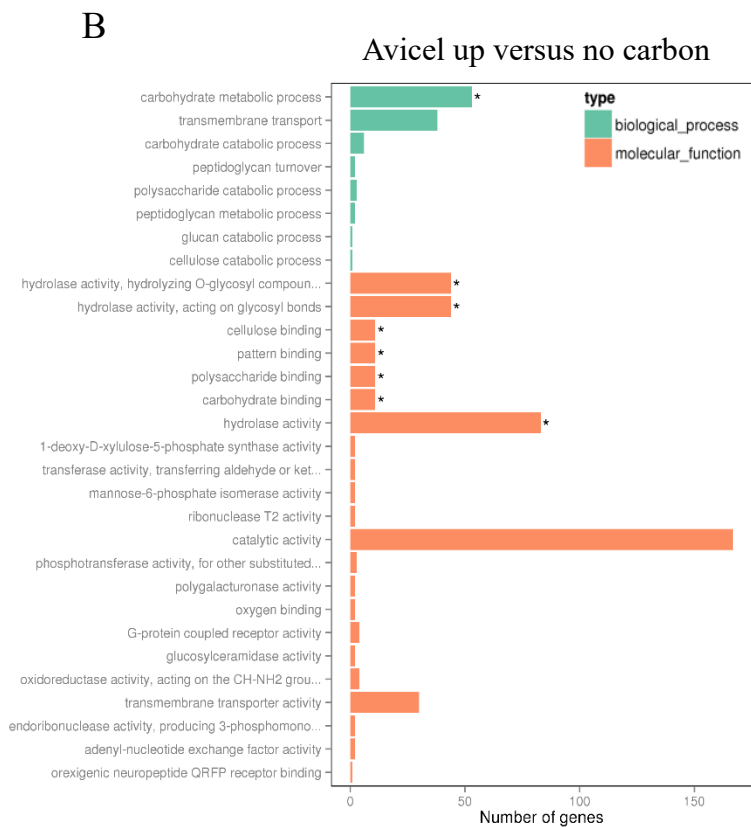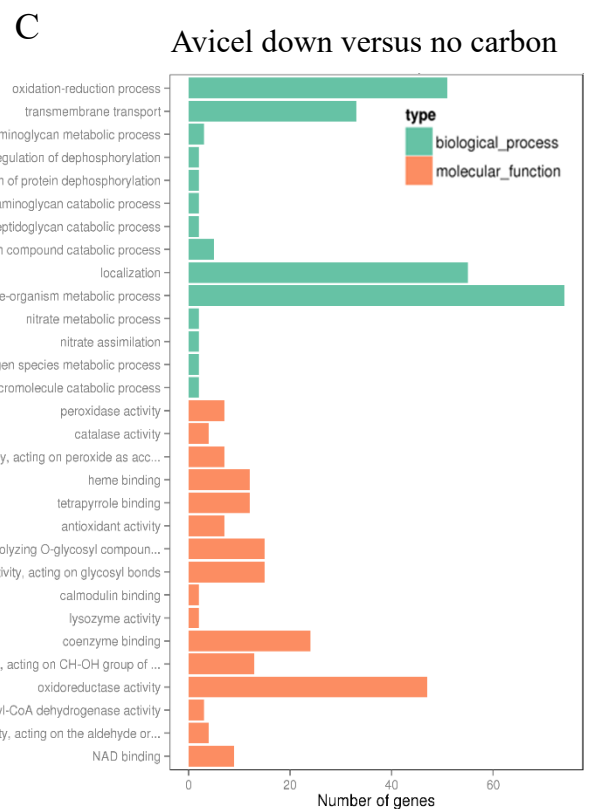

Supplement: Supplementary file 1 [file jof-07-00613-s001.zip › Supplementary Materials/Figure S2.pdf]

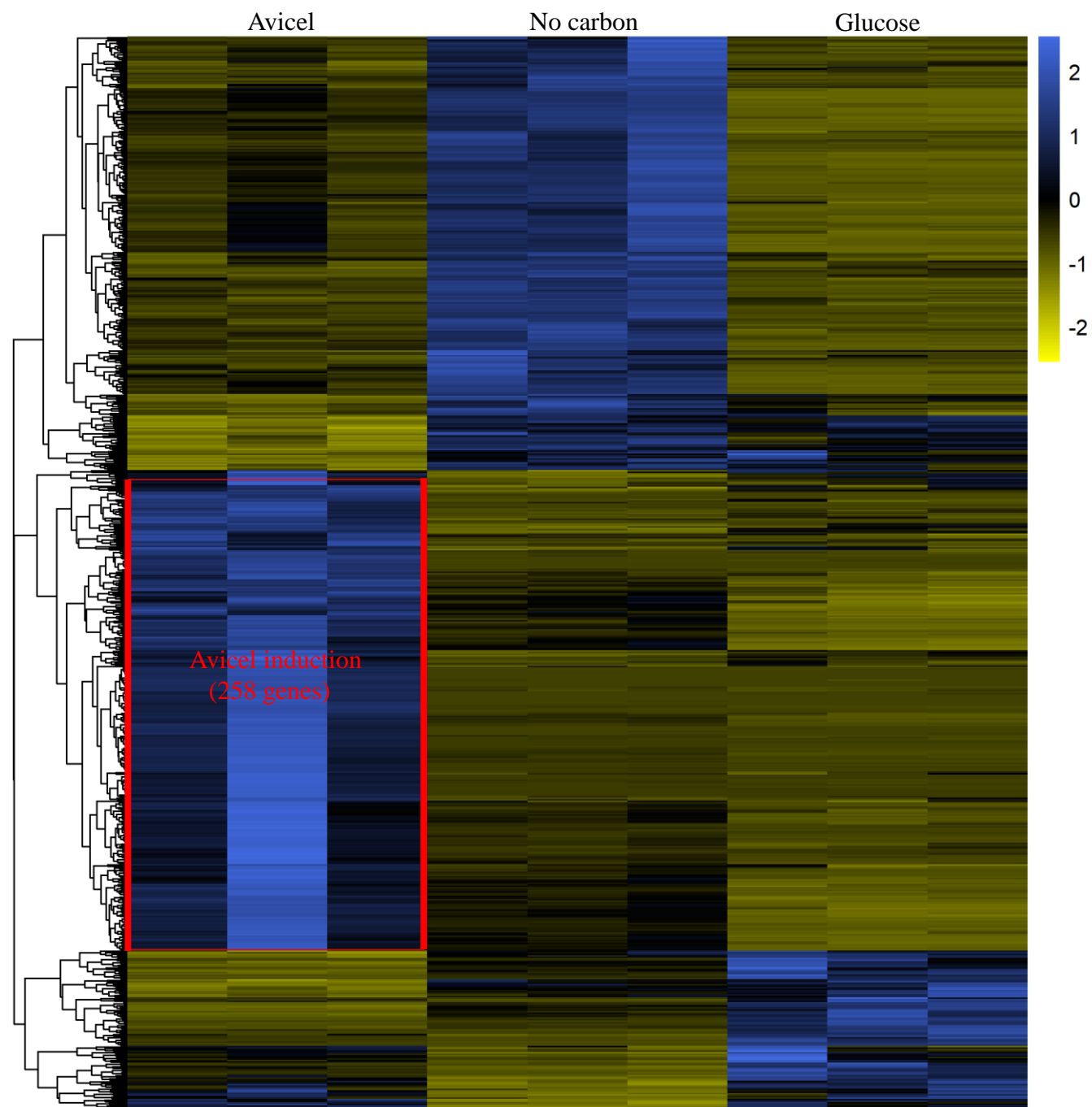

Supplement: Supplementary file 1 [file jof-07-00613-s001.zip › Supplementary Materials/Figure S3.pdf]

A

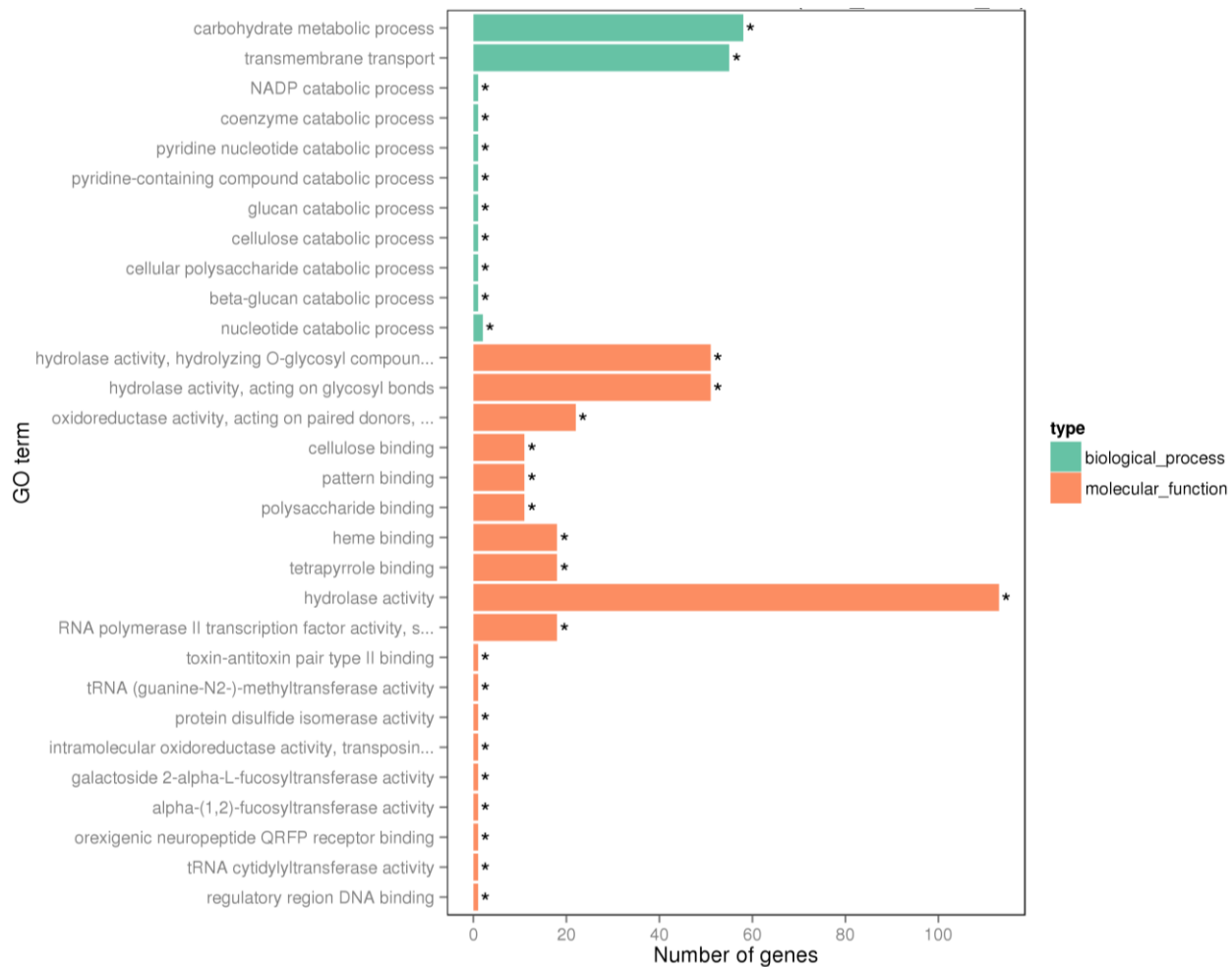

B

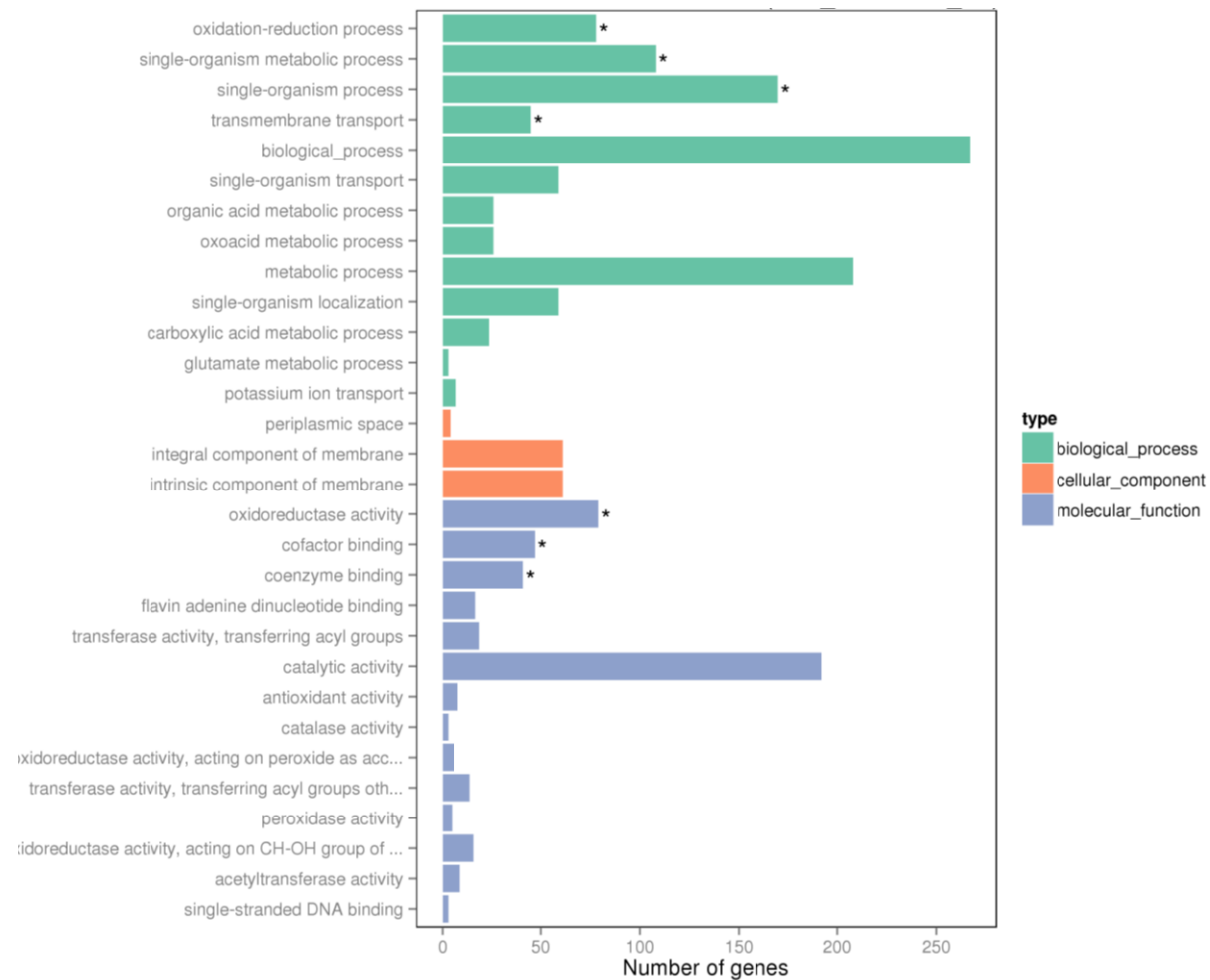

Supplement: Supplementary file 1 [file jof-07-00613-s001.zip › Supplementary Materials/Figure S4.pdf]
